# Impact of coronavirus disease 2019 (COVID-19) pandemic in hospital-acquired infections and bacterial resistance at an oncology hospital

**DOI:** 10.1017/ash.2023.148

**Published:** 2023-04-11

**Authors:** Patricia Cornejo-Juárez, Patricia Volkow-Fernández, Carla L. Vázquez-Marín, Nancy Álvarez-Romero, Bertha García-Pineda, Tania Chavez-Chavez, Diana Vilar-Compte

**Affiliations:** Infectious Diseases Department, Instituto Nacional de Cancerología (INCan), Mexico City, Mexico

## Abstract

**Objective:**

Hospital-acquired infection (HAI) rates were negatively affected by the the coronavirus disease 2019 (COVID-19) pandemic. We describe the incidence of HAIs, main pathogens, and multidrug-resistant organisms (MDROs) isolated in cancer patients before and during the pandemic.

**Design:**

This retrospective, comparative study included patients with HAIs. We compared 2 periods: the prepandemic period (2018, 2019, and the first 3 months of 2020) with the pandemic period (April–December 2020 and all of 2021).

**Setting:**

Instituto Nacional de Cancerología, a tertiary-care oncology public hospital in Mexico City, Mexico.

**Methods:**

Patients with the following HAIs were included: nosocomial pneumonia, ventilator-associated pneumonia (VAP), secondary bloodstream infection (BSI), central-line–associated bloodstream infection (CLBSI), and *Clostridioides difficile* infection (CDI). Demographic data, clinical characteristics, pathogens isolated, and MDRO data were included.

**Results:**

We identified 639 HAIs: 381 (7.95 per 100 hospital discharges) in the prepandemic period and 258 (7.17 per 100 hospital discharges) in the pandemic period. Hematologic malignancy was documented in 263 (44.3%) patients; 251 (39.2%) were in cancer progression or relapse. Nosocomial pneumonia was more frequent during the pandemic period (40.3% vs 32.3%; *P* = .04). Total episodes of VAP were not different between the 2 periods (28.1% vs 22.1%; *P* = .08), but during the pandemic period, the VAP rate was higher among COVID-19 patients than non–COVID-19 patients (72.2% vs 8.8%; *P* < .001). *Escherichia coli*, *Stenotrophomonas maltophilia,* and *Staphylococcus aureus* bacteremia cases were more frequent in the pandemic period. Extended-spectrum β-lactamases (ESBL)–*E. coli* was the only MDRO that occurred more frequently during the pandemic period.

**Conclusions:**

In cancer patients, nosocomial pneumonia was more frequent during the pandemic period. We did not observe a significant impact on other HAIs. MDROs did not significantly increase during the pandemic.

The coronavirus disease 2019 (COVID-19) pandemic increased healthcare demands and disrupted routine clinical-care practices. Healthcare personnel and infrastructure became insufficient in most hospitals. These factors could have negatively affected the prevention of hospital-acquired infections (HAIs). The increased number of patients under mechanical ventilation, many for prolonged periods with multiple comorbidities, steroid use, and other immunosuppression medication, could have increased some HAIs, such as ventilated-associated pneumonia (VAP).^1^ In contrast, the reinforcement of hand hygiene, isolation practices, and the increase in personal protective equipment (PPE) could have had a positive impact on reducing other HAIs.^2^ The rate of some HAIs, particularly those associated with multidrug-resistant organisms (MDROs), might have also benefited from all these measures, reducing their occurrence.^2^

In the early stages of the pandemic, little was known about COVID-19 hospitalizations and their effect on HAIs.^3^ Soon after, reports about the increase in secondary infections, higher HAIs rates, and unusual pathogens were documented.^3^ This elevated risk was exceptionally high in critically ill patients who needed mechanical ventilation and multiple invasive devices, with increased use of antibiotics, particularly broad-spectrum antibiotics with intense selective pressure for resistant pathogens and the occurrence of *Clostridioides difficile* enterocolitis.^4^

Patients with cancer have long been recognized to have an elevated risk for infections due to immunosuppression (by neoplasm itself and secondary to cancer therapy), in addition to the frequent use of long-dwelling invasive devices, extensive and complex surgeries, and continuous exposure to the healthcare environment.

In this study, we have described the rates and incidences of HAIs between 2 periods (prepandemic and pandemic) to estimate the impact of the COVID-19 pandemic on HAIs and the prevalence of antimicrobial resistance at an oncology center.

## Methods

This retrospective study was conducted at Instituto Nacional de Cancerología (INCan), a tertiary-care teaching institution for patients with cancer in Mexico City, Mexico. The study was approved by the INCan-Research Ethics Committee (REF/INCAN/CI/2022/0185).

The first COVID-19 patient in Mexico was diagnosed on February 27, 2020. On March 27, 2020, the first patient with cancer and COVID-19 was diagnosed at our Institution. Since March 23, 2020, INCan has functioned as a hybrid hospital for COVID-19 and non–COVID-19 patients.

During the first year of the pandemic, there was an increase in first-time patients because of referrals from other hospitals exclusively dedicated to COVID-19. Follow-up outpatient appointments decreased by 24%, and emergency room (ER) visits for cancer patients were 12.8% lower. Also, the number of patients with respiratory symptoms who required hospitalization grew by 12%.

At INCan, an exclusive COVID-19 ward for patients (both cancer patients and hospital employees) was installed. This area was divided into a COVID-19 area whose capacity varied from 6 to 15 beds over time, accordingly to the pandemic dynamics. The unit had a nurse for every 3 to 4 patients. The COVID-19 intensive care unit (ICU) had 8 beds for patients in critical condition, attended by an intensive care physician and 1 nurse per patient. All patients were evaluated and followed by the infectious disease team and fellows from other medical specialties.

Data pertaining to episodes of HAI were obtained from the database of the hospital-related infections surveillance program, and additional clinical and demographic patient data were collected from electronic medical records. All the microbiological information was obtained from the microbiology laboratory reports.

Antimicrobial identification was determined by mass spectrometry, especially matrix assisted laser desorption and ionization–time of flight mass spectrometry (MALDI-TOF MS, Microflex, Brueckner, Dover, NH) and susceptibility information was documented using Vitek-2 (bioMérieux, Hazelwood, MO) according to Clinical and Laboratory Standards Institute (CLSI) guidelines (2018–2021).

The following data were collected: age, sex, comorbidities, solid or hematological neoplasm, cancer status (divided into recent diagnosis, progression or relapse, and remission), current use of chemotherapy, radiotherapy, surgery, previous hospitalizations and use of antimicrobials, severe neutropenia (<500 cell/mm^3^), presence of a central venous catheter (CVC), admission to the ICU, length of ICU stay, presence of mechanical ventilation (MV), and days of MV.

We examined 5 HAIs in this study. Nosocomial pneumonia was defined using 3 criteria: (1) new and persistent, or progressive and persistent infiltrate, consolidation or cavitation in a chest imaging test, in patients hospitalized for ≥48 hours; (2) plus fever (>38º) or leukopenia (≤ 4000/m) or leukocytosis (≥ 12,000/mL) and, for adults aged ≥70 years, altered mental status with no other recognized cause; (3) and at least 2 of the following symptoms: new onset of purulent sputum or change in the character of sputum or increase in respiratory secretions, or increased suctioning requirements; new onset of worsening cough, or dyspnea, or tachypnea; rales or bronchial breath sounds; worsening gas exchange.^4^ Ventilator-associated pneumonia (VAP) was defined as a patient on mechanical ventilation for >2 consecutive days who developed pneumonia.^4^ Secondary bloodstream infection (BSI) was defined as a BSI that was thought to have originated from a site-specific infection at another body site.^5^ Central-line–associated bloodstream infection (CLABSI) was defined as a laboratory-confirmed BSI not related to an infection at another site that develops within 48 hours of central-line placement.^5^ *Clostridioides difficile* infection (CDI) was defined as either of the following criteria: positive test for toxin-producing *C. difficile* on an unformed stool specimens or patient with evidence of pseudomembranous colitis on gross anatomic or histopathologic exam.^6^

The primary pathogens isolated were divided into 3 categories: (1) gram-negative organisms, analyzing extended-spectrum β-lactamases (ESBL) or carbapenem-resistant (CR); (2) gram-positive organisms, including methicillin-resistant *Staphylococcus aureus* (MRSA) and vancomycin-resistant *Enterococcus faecium*; and (3) fungus (yeasts and molds).

### Statistical analysis

Descriptive statistics were analyzed comparing the prepandemic versus pandemic periods, using mean ± standard deviation, median and interquartile range (IQR) for continuous variables, and proportions for categorical variables. A subanalysis was performed between patients with COVID-19 and non–COVID-19 patients during the pandemic. For continuous variables, the *t* test or Mann-Whitney test was used as appropriate. A 2-sided *P* value ≤ .05 was considered statistically significant. All analyses were performed using Stata version 14 software (StataCorp, College Station, TX).

## Results

During the prepandemic period, there were 776 HAIs in 9,042 hospital discharges (rate, 7.95 per 100 hospital discharges). During the pandemic period, there were 995 HAIs in 11,402 hospital discharges (rate, 7.17 per 100 hospital discharges) (Supplementary Table).

We analyzed 639 patients with HAIs: 381 during the prepandemic period and 258 during the pandemic period. Overall, 52.6% of these patients were 336 male; the mean age was 49.9 ± 17.8 years; and patients were older in the pandemic period versus the prepandemic period (51.7 ± 17.5 vs 48.8 ± 18; *P* = .06). Diabetes mellitus, hypertension, and being overweight were more prevalent in patients during the pandemic period.

Related to neoplasm, 263 patients (44.3%) had a hematologic malignancy; 294 (46%) had recently been diagnosed with cancer and were starting anticancer treatment. Patients with HAIs with cancer progression were more frequent during the pandemic period versus the prepandemic period (32.2% vs 24.7%; *P* = .037). The use of antibiotics before the HAI episode was higher during the pandemic period than during the prepandemic period (48.3% vs 38.8%; *P* < .001). (Table 1).

**Table 1.** Clinical and Demographic Characteristics of All Patients Divided into Prepandemic and Pandemic Periods

Characteristic	Total (n=639), No. (%)^a^	Prepandemic Period (n=381), No. (%)^a^	Pandemic Period (n=258), No. (%)^a^	*P* Value
Age, mean y ±SD	49.9±17.8	48.8±18	51.65±17.5	.06
Sex, male	336 (52.6)	205 (53.8)	131 (50.8)	.451
Solid tumor	356 (55.7)	206 (54.1)	150 (58.1)	.309
Hematologic	263 (44.3)	175 (45.9)	108 (41.8)	
Oncology status				
Recent diagnosis	294 (46)	187 (49.1)	107 (41.5)	.058
Progression	177 (27.7)	94 (24.7)	83 (32.2)	.037
Relapse	74 (11.6)	49 (12.9)	25 (9.7)	.219
Remission	94 (14.7)	51 (12.4)	43 (16.6)	.250
Recent chemotherapy^b^	301 (47.2)	180 (47.2)	121 (47.1)	.931
Radiotherapy^c^	61 (9.6)	28 (7.4)	33 (12.8)	.021
Surgery^b^	225 (35.2)	139 (36.5)	86 (33.3)	.413
Previous hospitalizations^d^	246 (38.5)	153 (40.1)	93 (36.1)	.294
Previous antibiotics	275 (43)	136 (38.8)	139 (48.3)	<.001
Cephalosporins	99 (15.5)	45 (11.8)	54 (21)	.001
Carbapenems	149 (23.4)	87 (22.9)	62 (24.1)	.725
Fluoroquinolones	17 (2.7)	15 (4)	2 (1)	.021
TMP-SMX	47 (7.4)	27 (7.1)	20 (7.8)	.766
Penicillin	106 (16.6)	69 (18.2)	37 (14.4)	.208
Vancomycin	53 (8.3)	38 (10)	15 (5.8)	.061
Linezolid	13 (2)	6 (1.6)	7 (2.7)	.394
Comorbidities				
Diabetes mellitus	107 (16.8)	48 (12.6)	59 (22.9)	<.001
Hypertension	135 (21.1)	59 (15.5)	76 (29.4)	<.001
HIV	29 (4.5)	16 (4.2)	13 (5)	.699
HSCT	34 (5.3)	21 (5.5)	13 (5)	.858
Body mass index >26 g/m^2^	293 (45.9)	156 (40.9)	137 (53.1)	.002
Body mass index (g/m^2^), mean ±SD	24.6 ± 4.7	24.6 ± 4.7	25.9 ± 5	.0006
Severe neutropenia	195 (30.5)	113 (29.7)	77 (29.8)	.959
Days with neutropenia <500 cells/mm^3^, median d (IQR)	8 (3–17)	11 (4–19)	6 (3–13)	.012
Central venous catheter	465 (72.8)	165 (43.3)	62 (24)	<.001

Note. TMP-SMX, trimethoprim-sulfamethoxazole; HIV, human immunodeficiency virus; HSCT, hematopoietic stem cell.

a Units unless otherwise specified.

b During the last month.

c During the last 6 months.

d During the last 3 months.

We did not detect differences in the numbers of HAIs except for nosocomial pneumonia, which occurred more frequently during the pandemic period (40.3%) versus the prepandemic period (32.3%; *P* = .037). Although the VAP rate was not different between the 2 periods, when it was analyzed only in the pandemic, there were considerably more episodes in patients with COVID-19 than in non–COVID-19 patients (72.2% vs 8.8%, respectively; *P* < .001) (Table 2).

**Table 2.** Characteristics of Healthcare-Associated Infections (HAIs) and Microorganisms Isolated During the Two Study Periods

Characteristic	Total (n=639), No. (%)^a^	Prepandemic Period (n=381), No. (%)^a^	Pandemic Period (n=258), No. (%)^a^	*P* Value	Pandemic Period		
					Non–COVID-19 (n=204), No. (%)^a^	COVID-19 (n=54), No. (%)^a^	*P* Value
**No. of HAIs** ^**b**^							
1	543 (84.9)	331 (86.9)	212 (82.2)	.102	168 (82.3)	44 (81.5)	.849
2	82 (12.8)	43 (11.3)	39 (15.1)	.155	32 (15.7)	7 (13)	.830
3	14 (2.2)	7 (1.8)	7 (2.7)	.583	4 (2)	3 (5.6)	.161
**Type of HAI**							
Nosocomial pneumonia	227 (35.5)	123 (32.3)	104 (40.3)	.037	91 (44.6)	13 (24.1)	.006
VAP	164 (25.7)	107 (28.1)	57 (22.1)	.088	18 (8.8)	39 (72.2)	<.001
Secondary BSI	167 (26.1)	93 (24.4)	74 (28.7)	.227	66 (32.3)	8 (14.8)	.011
CLABSI	78 (12.2)	41 (10.8)	37 (14.3)	.175	31 (15.2)	6 (11.1)	.519
CDI	72 (11.3)	44 (11.5)	28 (10.9)	.784	25 (12.3)	3 (5.6)	.219
Length of hospital stay, median d (IQR)	20 (12–31)	19 (11–32)	20 (14–30)	.252	20 (13–29)	22 (17–33)	.01
ICU admission	211 (33.2)	139 (36.7)	72 (28)	.02	24 (11.8)	48 (88.9)	<.001
Length of ICU y, median d (IQR)	8 (3–15)	6 (3–13)	11 (4–17)	.013	5 (3–12)	17 (11–21)	<.001
Invasive mechanical ventilation	211 (33)	116 (30.5)	95 (36.8)	.09	49 (24)	46 (85.2)	<.001
Days of ventilation y, median d (IQR)	9 (4–17)	8 (2–15)	13 (5–19)	.002	6 (3–15)	18 (11–24)	<.001
Mortality at 72 h	48 (7.5)	27 (7.1)	21 (8.1)	.620	18 (8.8)	3 (5.6)	.580
Mortality at 30 d	221 (34.6)	122 (32)	99 (38.4)	.097	65 (31.9)	34 (62.9)	<.001
Mortality at 90 d	304 (47.6)	175 (45.9)	129 (50)	.312	90 (44.1)	39 (72.2)	.002

Note. VAP, ventilator-associated pneumonia; BSI, bloodstream infection; CLABSI, central-line–associated bloodstream infection; CDI, *Clostridioides difficile* infection; ICU, intensive care unit.

a Units unless otherwise specified.

b Total healthcare associated infections: 749 (341 in prepandemic and 408 in pandemic periods).

Overall mortality at 30 days occurred in 221 patients, 99 (38.4%) patients died during the pandemic period and 122 (32%) died in the prepandemic period (*P* = .09) During the pandemic period, mortality was significantly higher in COVID-19 patients (62.9%) versus non–COVID-19 patients (31.9%; *P* < .001). (Table 2).

We detected microbiology isolation in the cultures of 398 patients (62.4%): 211 (55.4%) in the prepandemic period and 187 (72.5%) in the pandemic period (*P* < .001). The main bacterium identified was *Escherichia coli* (*n* = 117 isolates); it occurred more frequently during the pandemic period vs the prepandemic period (24.8% vs 13.9%; *P* = .005), including ESBL–*E. coli*, which showed differences in both periods (60.9% vs 58.5%, respectively; *P* = .05). *Stenotrophomonas maltophilia* was more frequent in the pandemic period versus the prepandemic period (10.9% vs 4.5%; *P* = .001). Microorganisms that had not been isolated previously as a cause of HAI were identified in 6 patients during the pandemic period: 3 *Chryseobacterium indolegens* cases and 3 *Chryseobacterium gleum* cases. Other gram-negative bacteria did not show differences. Analyzing gram-positive bacteria, only *Staphylococcus aureus* was higher during the pandemic period versus the prepandemic period (8.9% vs 4.7%; *P* = .03). The rates of methicillin-resistant *S. aureus* (MRSA) were similar in both periods. Data are shown in Table 3.

**Table 3.** Main Pathogens Associated and Multidrug-Resistant Bacteria

Microorganisms	Total (n=639), No. (%)	Prepandemic Period (n=381), No. (%)	Pandemic Period (n=258), No. (%)	*P* Value
Gram negative	320 (86.7)	154 (40.4)	166 (63.3)	<.001
*Escherichia coli*	117 (18.3)	53 (13.9)	64 (24.8)	.005
Susceptible^a^	37 (31.6)	18 (34)	19 (29.7)	.170
ESBL^a^	70 (59.8)	31 (58.5)	39 (60.9)	.005
CR^*a*^	10 (8.5)	4 (7.5)	6 (9.4)	.213
*Klebsiella* spp	76 (11.9)	39 (10.2)	37 (14.3)	.115
Susceptible^a^	47 (61,9)	24 (61.5)	23 (62.2)	.214
ESBL^a^	27 (35.5)	14 (35.9)	13 (35.1)	.427
CR^a^	2 (2.6)	1 (2.6)	1 (2.7)	1
Other Enterobacteriacea	72 (11.3)	41 (10.8)	31 (12)	.622
Susceptible^a^	54 (75)	31 (75.6)	23 (74.2)	.728
ESBL^a^	18 (25)	10 (24.4)	8 (25.8)	.808
*Pseudomonas aeruginosa*	66 (10.3)	35 (9.2)	31 (12)	.248
Susceptible^a^	40 (60.6)	20 (5.2)	20 (7.8)	.243
CR^a^	26 (39.4)	15 (3.9)	11 (4.3)	.840
*Acinetobacter* spp	19 (3)	12 (3.1)	7 (2.7)	.816
Susceptible^a^	17 (89.5)	11 (2.9)	6 (2.3)	.804
CR^a^	2 (10.5)	1 (0.3)	1 (0.4)	1
*Stenotrophomonas maltophilia*	45 (7)	17 (4.5)	28 (10.9)	.001
*Chryseobacterium* spp	6 (0.9)	0	6 (2.3)	N/A
Other non-fermentative	6 (0.9)	0	6 (2.3)	N/A
Gram-positive cocci	91 (14.2)	46 (12)	45 (17.4)	.056
*Staphylococcus aureus*	41 (6.4)	18 (4.7)	23 (8.9)	.033
MRSA	6 (14.6)	2 (11.1)	4 (17.4)	.227
Coagulase-negative *staphylococcus*	31 (4.9)	18 (4.7)	13 (5)	.853
*Enterococcus* spp	17 (2.7)	8 (2.1)	9 (3.5)	.321
*Streptococcus* spp	16 (2.5)	9 (2.4)	7 (2.7)	.800
*Aspergillus* spp	31 (4.8)	16 (4.2)	15 (5.8)	.355
*Candida albicans*	13 (2)	6 (1.6)	7 (2.7)	.394
Non–*C. albicans*	20 (3.1)	12 (3.1)	8 (3.1)	1

Note. ESBL, extended-spectrum β-lactamase; CR, carbapenem resistant; MRSA, methicillin-resistant *Staphylococcus aureus*; N/A, not available.

a Percentages were obtained from the total number of each bacterium.

Patients who received antibiotics for the HAI event were similar in the 2 periods (89% in the prepandemic period and 87.6% in the pandemic period). However, when evaluating only the pandemic period, more patients with COVID-19 (98.1%) received antibiotics compared with non–COVID-19 patients (84.8%; *P* = .004). When specific antibiotics were examined, several were used at higher rates during the pandemic period: cephalosporins (39.9% vs 19.9%; *P* < .001), fluoroquinolones (10.1% vs 4.5%; *P* = .005), linezolid (16.7% vs 6.6%; *P* < .001), and SMX-TMP (21.3% vs 14.7%; *P* = .03). In contrast, the use of some antibiotics decrease during the pandemic period: piperacillin-tazobactam (30.2% vs 23.3%; *P* = .05) and macrolides (17.1% vs 7.8%; *P* < .001) (Table 4).

**Table 4.** Use of Antibiotics and Antifungals Related with to Healthcare-Associated Infections (HAIs) During the Prepandemic and Pandemic Study Periods

Characteristic	Total (n=639), No. (%)	Prepandemic Period (n=381), No. (%)	Pandemic Period (n=258), No. (%)	*P* Value
Received antibiotics	565 (88.4)	339 (89)	226 (87.6)	.592
Cephalosporins	181 (28.3)	78 (19.9)	103 (39.9)	<.001
Days of cephalosporins, mean ±SD	6.1 ± 3.7	5.5 ± 3.6	6.4 ± 3.8	.204
Piperacillin/tazobactam	175 (27.4)	115 (30.2)	60 (23.3)	.05
Days of piperacillin/tazobactam, mean ±SD	5.8 ± 3	6.3 ± 3	4.9 ± 2.8	.003
Aminopenicillins	58 (9.1)	35 (10)	23 (8)	.384
Days of aminopenicillins, mean ±SD	8.3 ± 6.4	9.3 ± 6.9	7 ± 4.7	.209
Carbapenems	424 (66.4)	246 (64.6)	178 (69)	.245
Days of carbapenems, mean ±SD	9.5 ± 8.9	10 ± 7.1	8.9 ± 6.3	.09
Amikacin	28 (4.4)	16 (4.2)	12 (4.7)	.844
Days of amikacin, mean ±SD	5.8 ± 4.9	4.9 ± 6.3	6.7 ± 2.9	.342
Fluoroquinolones	43 (6.7)	17 (4.5)	26 (10.1)	.005
Days of fluoroquinolones, mean ±SD	6.2 ± 3.9	7.5 ± 4	5.4 ± 3.7	.09
Macrolides	85 (13.3)	65 (17.1)	20 (7.8)	<.001
Days of macrolides, mean ±SD	6.1 ± 3.2	6.4 ± 3.4	5.1 ± 2.1	.121
Vancomycin	201 (31.5)	129 (33.9)	72 (27.9)	.119
Days of vancomycin, mean ±SD	6.3 ± 5.7	6.6 ± 4.7	5.9 ± 4.2	.293
Linezolid	68 (10.6)	25 (6.6)	43 (16.7)	<.001
Days of linezolid, mean ±SD	8.3 ± 6.6	8.8 ± 4.9	7.9 ± 7.5	.615
SMX/TMP	111 (17.4)	56 (14.7)	55 (21.3)	.03
Days of TMP-SMX, mean ±SD	10.2 ± 7.9	10.9 ± 6.4	9.5 ± 9.1	.352
Metronidazole	70 (11)	46 (12.1)	24 (9.3)	.271
Days of metronidazole, mean ±SD	7.4 ± 4.5	7.8 ± 4.4	6.5 ± 4.7	.243
Colistin	23 (3.6)	12 (3.1)	11 (4.3)	.518
Days of colistin, mean ±SD	7.2 ± 4.9	7 ± 4.3	7.5 ± 6.3	.841
Amphotericin	64 (10)	38 (10)	26 (10.1)	.965
Days of amphotericin, mean ±SD	11.2 ± 7	12.2 ± 7.7	9.7 ± 5.8	.156
Caspofungin	71 (11.1)	43 (11.3)	28 (10.9)	.864
Days of caspofungin	9.5 ± 5.9	8.9 ± 6.2	10.4 ± 5.4	.325
Voriconazole	67 (10.5)	45 (11.8)	22 (8.5)	.183
Days of voriconazole, mean ±SD	16.4 ± 15	16.7 ± 16	16.1 ± 12.8	.906
Itraconazole or fluconazole	58 (9.1)	33 (8.7)	25 (9.7)	.657
Days of itraconazole or fluconazole, mean ±SD	8.8 ± 5.7	8.2 ± 4.7	9.8 ± 6.8	.272

Note. TMP-SMX, trimethoprim-sulfamethoxazole.

## Discussion

In this study, we compared the incidence of HAI during the first 2 years of the COVID-19 pandemic with the 2 previous years. HAIs are a quality-of-care marker in different settings with well-established preventive measures. During the pandemic, many hospitals faced overburdened healthcare services, and conversions led to suboptimal care of non–COVID-19 patients in ambulatory and in-hospital settings. Hospitals faced an enormous challenge trying to accomplish preventive policies for HAI, partly due to the lack of medical supplies in some settings, understaffing, and overworked healthcare personnel, which together probably had a substantial impact on the increase of HAIs.^7^

The clinical and demographic characteristics of cases described in this cohort during the pandemic included those described repeatedly in multiple studies: older age, diabetes mellitus, hypertension, and overweight. We analyzed specific cancer-related characteristics and found that active cancer was more common during the pandemic. This difference has been linked to multiple factors, such as fear of patients going to the hospital, reduced medical attention in many in-hospital and outpatient services, and some hospitals converted exclusively for COVID-19 attention.^8–10^

In this study, for the whole group, nosocomial pneumonia increased in patients during the pandemic period. When we analyzed this period, the nosocomial pneumonia rate was higher in non–COVID-19 patients; contrary to what occurred in patients with COVID-19, in whom VAP was significantly more frequent, as has been reported in multiple studies.^11^ VAP in COVID-19 patients was not only related to prolonged stays in the ICU and mechanical ventilation. Still, it was also associated with microaspirations, partly caused by prone positioning and acute respiratory distress syndrome, more steroid use, higher cumulative dosages, and more frequent use of JAK inhibitors.^11^ These factors favor impairment of immune cell function, damage to the alveolar membrane, and dysbiosis of the respiratory and digestive microbiota.^12,13^ In addition, several infection control measures for nosocomial pneumonia and VAP were not consistently followed in patients requiring intubation and pronation, in addition to overcrowding the ICU and converting different hospital wards into critical care units.^12^

In contrast, higher compliance with hand hygiene, environmental cleaning, patient isolation, and PPE during the pandemic probably helped maintain similar CDI and CLABSI rates in both periods.^12,14^ These findings also were observed in our series, in which CDI was not different between study periods; in fact, secondary BSI was lower during the pandemic periods (14.8%) in comparison to the prepandemic period (32.3%; *P* = .01).

In the first months of the COVID-19 pandemic, there was an excessive prescription of antimicrobials, particularly fluoroquinolones and third-generation cephalosporins. These were reported in 70%–74% of cases, mainly in those patients who developed the severe form of the disease, even in the absence of microbiologic isolation or strong suspicion of bacterial infection.^4,15–17^ Approximately 8% of COVID-19 patients were diagnosed with secondary infections, which were more frequent in the ICU (8%–14%) compared with patients from other wards (4%–6%).^17^ In our study, we found elevated use of antibiotics, with no difference between periods (89% in the prepandemic period and 87.6% in the pandemic period), probably related to the type of patients treated at our hospital, with different levels of immunosuppression and neutropenia, in whom antibiotics are started empirically.

Gram-negative isolates are the microorganisms most frequently isolated in COVID-19 patients with secondary infections.^16,18,19^ Other reports have described *Acinetobacter baumannii* and MRSA as frequent pathogens.^19,20^ We identified an increase in the isolation of *E. coli*, *S. maltophilia*, and *S. aureus*.

Increases in MDROs has been described in several reports during the COVID-19 pandemic as pathogens associated with HAIs: 75.5% in CR–*K. pneumoniae*, 91.2% in CR–*A. baumannii*, and 50% in MDR- *S. maltophilia*,.^21^ In this study, we found no essential change in bacterial resistance between the 2 periods, except for ESBL–*E. coli* (60.9% in the pandemic period versus 58.5% in the prepandemic period; *P* = .05). Also, bacteria that had never been isolated in our hospital as a cause of VAP were identified, including *Chryseobacterium indologenes* and *C. gleum,* which were isolated only in patients with COVID-19.

Fungal infections, especially *Aspergillus* spp and *Candida* spp, have been associated with COVID-19.^21,22^ In this study, the isolation of *Aspergillus* spp was documented in 4.8% of patients, without differences in the 2 periods; This rate was lower than those of other reports in Mexico during the first months of the pandemic, with prevalences between 9.7% and 19.3%.^23,24^ *Candida* spp were also less frequent as a cause of infection (3.1%) than in other reports (14.4%).^25^

The overall 30-day mortality was 34.6%, similar to that described in other studies during the pandemic (30%–40%).^16,22^ But when we divided the patients seen during the pandemic with or without COVID-19, the mortality rate was significantly higher among patients with COVID-19 infection (62.9%). This could have been related to neoplasm; 39.2% of patients were in cancer progression or relapse and 46% had recently been diagnosed and were starting the cancer treatment.

This study had several limitations. Data were collected only in a single institution. Because it is an oncological center, the general mortality may have been higher than that reported in other series. Due to its retrospective nature and the emergency of COVID-19, some information bias probably occurred. Nevertheless, this series has provided a local view of conditions before the COVID-19 pandemic and throughout the first 2 years of the pandemic at an oncological hospital that functioned hybrid from the beginning of the pandemic.

In this study, we showed that although hospital functioning was significantly disrupted by the need to care for COVID-19 patients, the number of patients with newly diagnosed cancer did not decrease.

In summary, in patients with cancer, nosocomial pneumonia was more frequent during the pandemic, and VAP was more frequent in patients with COVID-19. We detected an increase in the isolation of unusual bacteria during the pandemic, but the only significant MDR increase was documented for ESBL–*E. coli*. A regular review of surveillance data, continuous observation, and analysis of infection control policies and practices are critical for hospitals to identify gaps in prevention to address any increase in HAIs during emergencies like that posed by the ongoing COVID-19 pandemic.

## Supplementary material

For supplementary material accompanying this paper visit http://doi.org/10.1017/ash.2023.148. click here to view supplementary material
