# Supplementary material for: Impact of coronavirus disease 2019 (COVID-19) pandemic in hospital-acquired infections and bacterial resistance at an oncology hospital
Source: Antimicrob Steward Healthc Epidemiol. 2023 Apr 11;3(1):e70. doi: 10.1017/ash.2023.148 (PMC10127242; doi:10.1017/ash.2023.148)
Supplement: Supplementary file 1 [file S2732494X23001481sup001.docx]

Supplementary material. Hospital and infections related data before and during the COVID-19 pandemic

| Characteristics | Pre-pandemic^a^ | | Pandemic^b^ | |
| --- | --- | --- | --- | --- |
|  | Total | Monthly average | Total | Monthly average |
| Patient newly diagnosed with cancer | 6683 | 445 | 9685 | 461 |
| Hospital discharges | 9042 | 602.8 | 11402 | 542.5 |
| HAI episodes | 776 | 51.7 | 995 | 47.3 |
| Infected patients | 648 | 43.2 | 831 | 39.5 |
| Total of hospital-stay (days) | 50090 | 3339 | 66381 | 3161 |
| HAI Incidence rate /1000 days | 14.6 | 15.5 | 14.3 | 14.5 |
| Surgeries performed | 4549 | 303 | 4832 | 230 |

^a^Pre-pandemic- 27 months (2018, 2019, the first three months of 2020). ^b^Pandemic: 21 months (from April 2020 to December 2021).
